# The Interplay of Dysregulated pH and Electrolyte Imbalance in Cancer

**DOI:** 10.3390/cancers12040898

**Published:** 2020-04-07

**Authors:** Khalid O. Alfarouk, Samrein B. M. Ahmed, Ahmed Ahmed, Robert L. Elliott, Muntaser E. Ibrahim, Heyam S. Ali, Christian C. Wales, Ibrahim Nourwali, Ahmed N. Aljarbou, Adil H. H. Bashir, Sari T. S. Alhoufie, Saad Saeed Alqahtani, Rosa A. Cardone, Stefano Fais, Salvador Harguindey, Stephan J. Reshkin

**Affiliations:** 1 Alfarouk Biomedical Research LLC, Temple Terrace, FL 33617, USA; 2 American Biosciences Inc., New York, NY 10913, USA; dwales@americanbiosciences.com; 3 Hala Alfarouk Cancer Center, Khartoum 11123, Sudan; 4 College of Medicine, University of Sharjah, P. O. Box 27272 Sharjah, UAE; samahmed@sharjah.ac.ae; 5 Department of Oesphogastric and General Surgery, University Hospitals of Leicester, Leicester LE5 4PW, UK; Ahmed.ahmed@doctors.org.uk; 6 The Sallie A. Burdine Breast Foundation, Baton Rouge, LA 70806, USA; drrobertelliott@cox.net; 7 Institute of Endemic Diseases, University of Khartoum, Khartoum 11111, Sudan; mibrahim@iend.org (M.E.I.); Derma55@yahoo.com (A.H.H.B.); 8 Department Pharmaceutics, Faculty of Pharmacy, University of Khartoum, Khartoum 11111, Sudan; Heyam57@hotmail.com; 9 College of Dentistry, Taibah University, Al-Madinah Al-Munwarah 42313, Saudi Arabia; ibrahim_germany@hotmail.com; 10 College of Pharmacy, Qassim University, Buraydah 51452, Saudi Arabia; aaljarbou@yahoo.com; 11 Department of Clinical Laboratory Sciences, Faculty of Applied Medical Sciences, Taibah University, Al-Madinah Al-Munwarah 42353, Saudi Arabia; saritalal1@hotmail.com; 12 Clinical Pharmacy Department, College of Pharmacy, Jazan University, Jazan 45142, Saudi Arabia; saad6620@hotmail.com; 13 Department of Biosciences, Biotechnologies, and Biopharmaceutics, University of Bari, 90126 Bari, Italy; rosaangela.cardone@uniba.it (R.A.C.); stephanjoel.reshkin@uniba.it (S.J.R.); 14 Department of Oncology and Molecular Medicine, National Institute of Health, Viale Regina Elena, 299, 00161 Rome, Italy; stefano.fais@iss.it; 15 Institute for Clinical Biology and Metabolism, Postas 13, 01004 Vitoria, Spain; salvaszh@telefonica.net

**Keywords:** cancer, pH, electrolytes, metabolism

## Abstract

Cancer cells and tissues have an aberrant regulation of hydrogen ion dynamics driven by a combination of poor vascular perfusion, regional hypoxia, and increased the flux of carbons through fermentative glycolysis. This leads to extracellular acidosis and intracellular alkalinization. Dysregulated pH dynamics influence cancer cell biology, from cell transformation and tumorigenesis to proliferation, local growth, invasion, and metastasis. Moreover, this dysregulated intracellular pH (pHi) drives a metabolic shift to increased aerobic glycolysis and reduced mitochondrial oxidative phosphorylation, referred to as the Warburg effect, or Warburg metabolism, which is a selective feature of cancer. This metabolic reprogramming confers a thermodynamic advantage on cancer cells and tissues by protecting them against oxidative stress, enhancing their resistance to hypoxia, and allowing a rapid conversion of nutrients into biomass to enable cell proliferation. Indeed, most cancers have increased glucose uptake and lactic acid production. Furthermore, cancer cells have very dysregulated electrolyte balances, and in the interaction of the pH dynamics with electrolyte, dynamics is less well known. In this review, we highlight the interconnected roles of dysregulated pH dynamics and electrolytes imbalance in cancer initiation, progression, adaptation, and in determining the programming and reprogramming of tumor cell metabolism.

## 1. Introduction

In most cells, the utilization of glucose by the cell yields energy in the form of ATP and other molecules, ultimately ending in CO_2_ and H^+^. Under aerobic conditions, O_2_ binds a proton to form H_2_O; therefore, O_2_ acts as an intracellular detoxifying molecule [1]. During transient low oxygen conditions and in only some relatively adaptable tissues (e.g., skeletal muscle but not in the brain nor heart) glycolysis produces lactate (converted from pyruvate), which is then translocated (extruded), via proton-linked monocarboxylate transporters (MCTs), creating an acidic extracellular pHe [2,3,4,5,6,7]. This transient shift in the metabolic program under oxygen scarcity is termed the “Pasteur effect” [8]. Chronic endurance training increases muscle blood flow capacity (BFC) through the promotion of vascularity (remodeling of the arterial tree and/or increasing vascular resistance), and so increases oxygen levels [9,10,11]. While intermittent hypoxia leads to a disastrous effect on cell fate ending in cell death, skeletal muscle cells have adapted [12] by acquiring aberrant biology through disruption and/or atrophy of mitochondria [13]. The Pasteur effect could be seen as an economical cost during a deficit of O_2_ because it represents the cytoplasmic utilization of glucose efficiently, ending up in the formation of lactate. In conclusion, in normal tissue, oxygen is generally a critical component in the cellular detoxifying process.

In the 1920s, Otto Warburg observed that most cancer cells could perform glycolysis in the presence of oxygen with a dominant production of lactate, which later came to be known as the “Warburg effect” and is considered to be a cancer hallmark [2,14]. The consequence of the upregulated glycolysis is creating alkaline pH_i_ conditions inside the cell and an acidic extracellular pHe [1,15]. The pH dysregulation observed in cancer cells helps to drive various tumor hallmark processes that are highly sensitive to even minimum pHi variations such as cancer initiation, progression, adaptation, proliferation, migration, and the reprogramming of tumor cell metabolism. Furthermore, many effects on biologic processes of a cell are generated as a result of pH-sensitive functions such as the regulation of protein expression, the affinity of proteins to bind with a ligand as well as the activity of ion channels/enzymes that are important to maintain electrolyte dynamics in normal cells and create an electrolyte imbalance within the tumor cell.

In this review, we shall start out with a general discussion of the dynamics, regulation, and role of intracellular and extracellular pH in cancer processes followed by a discussion of what is known about the interaction of these pH dynamics with the dynamic and regulation of important cellular electrolytes such as sodium, bicarbonate, calcium, potassium, and chloride.

## 2. Extracellular Acidity and pH Sensors

Transmembrane pH sensors provide crucial information about the signal pathways that transduce the acidic extracellular pH ‘signal’ towards the reprogramming of genes and cancer processes within tumor cells [16]. G protein-coupled receptors (GPCRs), such as GPR4, 65 and 68, respond to increased extracellular proton levels via protonation of histidine residues in their extracellular regions [17] which then transduces the signals by various G proteins and several intracellular pathways such as phospholipase C (PLC) as well as adenylyl cyclase. There are also some non-G protein-coupled receptors such as TRPV1 (i.e., transient receptor potential V1) as well as ASIC1 (i.e., acid-sensing ion channel 1) that are activated by the acidic extracellular pH [18]. Recently in prostate and breast cancer cells exposed to the acidic environment, it has been reported that as these channels permit the influx of calcium followed by the activation of nuclear factor-κB (i.e., NF-κB) [19,20].

Furthermore, intracellular pH sensing is now known to play essential roles. Indeed, due to the nature of His and Arg residues to carry the charge, intracellular pH sensing may occur directly as a result of changes in the level of protonation of proteins involved in various signaling mechanisms. An example of this is the activity of most enzymes in the glycolysis pathway, which are pH-sensitive with optimum activity at alkaline pH and mutations in these residues can alter the overall activity of the enzymes (see below).

## 3. The Impact of pH on the Proliferation and the Survival of Cancer Cells

Alkaline intracellular pHi was considered by Pouyssegur and colleagues [21] to be not obligatory but rather permissive to increase the proliferation of cells, and pHi-dependent changes in cell proliferation have been confirmed by a recent study [22]. Various studies have suggested that the proliferative response based on pHi is dependent on the increased/decreased activity of transporters such as the sodium and hydrogen exchanger 1 (NHE1) [23], bicarbonate transporters driven by sodium [24,25] as well as the H^+^/K^+^-ATPase proton pump [26]. In the cell cycle regulation of a normal cell, when in interphase, the cells follow a subsequent pH increase until they reach the end of the S phase, promoting the transition to the G_2_/M phase. Any blockage of the increasing pH causes attenuation of the level of cyclin B as well as maintaining inhibitory phosphorylation of Cyclin-Dependent Kinase 1 (Cdk1-pTyr15). It is still difficult to determine the reason behind the regulation of cell cycle progression by the presence of a high alkaline pHi in cancer cells [21]. The study on the fibroblast cell lines has suggested an increase in the level of cyclin B, along with the level of Cell Division Cycle 25 (Cdc25) phosphatases (which causes dephosphorylation of inhibitory of Cdk1 Cdk1-pTyr15) as well as a reduction in the Wee1 kinase level (which causes phosphorylation of Cdk1- Tyr15) due to a constant increase in pHi [27]. Additionally, such changes in expression result in increasing the expression of active Cdk1, which causes promotion of the entry into the G2/M phase.

On the other hand, apoptotic cells have more acidic intracellular pH [28,29], and cytosolic alkalinization through activation of the Na^+^/H^+^ exchanger was found to suppress apoptosis through the inactivation of a pH-dependent endonuclease [29]. This acidification of the cytosol has been demonstrated to be an early event that regulates caspase activation in the mitochondrial pathway for apoptosis [29,30]. A reduction in the levels of mitochondrial pHi has been found during the apoptosis response mediated by death receptors [31], and this death-receptor acidification of pHi depends upon the caspases, which further cause DNA fragmentation [32]. It has been suggested that a reduced pHi is the sign of early caspase activation during the mitochondria-mediated apoptosis since a reduction in pH causes the mitochondria to release cytochrome C [33]. In support of this, it was observed that caspases are activated through cytochrome c, and this process needs cytosolic pH in the range between 6.3 and 6.8 [30].

## 4. Tumor Metabolism

In the 1920s, Otto Warburg observed that most cancer cells could perform glycolysis in the presence of oxygen with a dominant production of lactate, which later came to be known as the “Warburg effect” [2,14]. While the cytoplasmic utilization of glucose occurs only where mitochondria poorly function or are unable to function [1], it has been observed that many cancer cells can balance their metabolism between glycolysis and mitochondrial pathways [34,35,36,37]. Indeed it has been observed that some cancer cells can also rely on the lactate produced by other strongly glycolytic cancer or stromal cells known as “reversed Warburg effect” in that the ‘oxidative’ tumor cells are utilizing lactate produced by another glycolytic (Warburg) tumor cell [38]. Therefore, we proposed that cancer is an integrated metabolic ecosystem [8], and such metabolic diversity is governed by reaction-diffusion kinetics as a function of tumor vascularization [39] as well as the capability of tumors to wash out their metabolic waste products [40]. Intracellular alkalinity supports all the glycolytic steps to provide ATP and NADH [1] as well as maintaining the redox state of the cell through the Pentose Phosphate Pathway (PPP) pathway to support cellular proliferation and diminish cell death [2].

The alkaline pH_i_ is the result of the activation of many proton exchangers/ion channels that is acting together to make the extracellular milieu acidic [41]. This acidic pH_e_ confers an “evolutionarily advantageous” cancer fitness [41] by supporting tumor growth via: (i) diminishing growth of the normal cells and so provide more space and relative abundance of nutrients, e.g., glucose, (ii) blunting the immune system [41], (iii) supporting metastatic transformation [41], and (iv) the acidic pH ionizes the weak basic chemotherapeutics agents and so reduces their entrance to the cell through lipid bilayers [42].

This type of programming and reprogramming of metabolism is beneficial to cancer cells as it increases their resistance to hypoxia. Hence, the nutrients are rapidly converted into biomass, thereby increasing cell proliferation, as well as providing protection against the damaging ROS produced by mitochondria. However, it has been found that in several types of cancer, there is an increase in glucose uptake as well as the production of lactic acid. Glycolytic flux depends partly on the activity of glycolytic enzymes having a strong alkaline pH sensitivity, such as lactate dehydrogenase A (LDHA) and Phosphofructokinase 1(PFK-1) [1].

LDHA regulates the conversion of pyruvate into lactate and is associated with a high level of aggressiveness in terms of its expression/activity in metastatic cancers. Moreover, tumor growth will be suppressed upon inhibition of LDH [43,44]. Indeed, in pancreatic tumors, the post-translational acetylation of Lys5 tends to reduce LDHA activity and, hence, reduces the growth of tumors due to the replacement of endogenous LDHA with a mutant of acetylation–mimetic [45]. LDHA activity is also regulated by the post-translational modification caused by protonation/deprotonation and, physiologically, due to a higher, alkaline pHi [46]. In LDHA, potential sensing regions of pH such as Asp140 which have been identified and form an electromagnetic bond in the catalytic site with the His192 in its backbone and the other region consists of Lys131 which is exposed to solvent and also contain a residue network at the interface of the tetramer, i.e., Arg170, His180 and 185 have been identified with the help of a computational program known as pHinder. The residues in the sensing regions can modify the values of pKa values shifting in an upward or downward direction in terms of physiological range. The details of the molecular mechanism of pH sensing with the help of LDHA are still undetermined [47].

Highly proliferating cancer cells show a characteristic feature of enhanced lactate activity; however, in glucose metabolism, the pathways that lead to an upstream flow of carbon is still not clear. Furthermore, it is known that in glycolysis, the activity of the first-rate limiting enzyme, i.e., PFK-1 is sensitive to levels of pH depicting more than a tenfold increase between the pH range of 7.0–7.4 although several studies have suggested variability in the PFK-1 role in cancer [48].

The enhanced expression of the protein PFK-1 in several cancers has suggested the high activity of PFK-1 is related to enabling the phenotypes of cancer cells [49]. Furthermore, PFK-1 glycosylation also shows high activity in cancer [49]. In addition to that, it was shown in several human cancers that various somatic mutations found in PFK-1 have the ability to inhibit its enzyme activity [50]. A PFK-1 platelet isoform has been used to understand the dysregulated pH dynamics of PFK-1 activity as a crystal structure. His208 was found to be the pH sensing residue based on the crystal structure of PFK-1, its estimated pKa, and the simulations based on the molecular dynamics [50].

*KRAS* (K-ras or Ki-ras) is a gene that governs cell signaling. The *KRAS* gene encodes an approximately 21 kDa small GTPase, that cycles between the active guanosine triphosphate-bound form (GTP) and the inactive guanosine diphosphate bound form (GDP) [51]. Normally, it controls cell proliferation. However, if mutated, the negative signaling perturbated, and so, the cells proliferate continually. Oncogenic activation of *KRAS* can affect several cellular processes that regulate morphology, proliferation, metabolism, motility, and survival via the activation of many pathways, such as the MAPK and PI3K/AKT/mTOR pathways [52,53]. KRAS expression stimulates the Warburg effect through enhancement of the expression many genes, including the gene responsible for the expression of the glucose transporter-1 (GLUT1) and several rate-limiting glycolytic enzymes, including hexokinase and lactate dehydrogenase. Also, KRAS expression supports the Hexosamine Biosynthesis pathway (HBP) and Pentose Phosphate Pathways (PPP); and so, pooling the DNA building blocks and inhibiting the cell death pathway (e.g., apoptosis) through the modulation of the NADP^+^/NADPH ratio [54,55,56].

Several treatment modalities have been adopted to interfere with glycolysis, e.g., some pharmacological agents used to treat cancer via restoration of the mitochondrial function to support programmed cell death (apoptosis); some of these agents include Proton Pump Inhibitors (PPIs), Hydroxycitrate, NaHCO_3_, Lipoic acid, and could be extended to use some of the natural extracts, e.g., Fermented wheat germ extract FWGE [14,57,58,59,60,61,62].

## 5. Effect of pH on Tumor Metabolism

Various studies have suggested the importance of tumor environmental acidification on metabolic programming [39,41,63,64,65,66]. Although, it was identified that the cellular mechanisms that vent protons are limited in their action, and the cancer cells reprogram their metabolism uniquely to fulfill the challenging environment. The whole process is regulated by an efficiently working mechanism that tends to maintain the pHi of tumor cells within the alkaline range, even in the presence of an acidic environment [67]. The regulation of pH in tumor cells is carried out with the help of classical models such as hydrogen extrusion or bicarbonate cytoplasmic buffering [68].

Due to alterations in cancer cell metabolism, tumor cells secrete a massive amount of lactate into the tumor microenvironment. The adaptations made by tumor metabolism in response to acidosis of the tumor microenvironment are linked with the development of a lactate gradient in hypoxic cancer cells. Lactate is a product of glycolysis released from the cell via the MCT4, a process that is referred to as metabolic symbiosis as it can then be taken up consumed by other cancer cells [69,70,71]. Various studies have recognized HIF2α as a critical regulator which helps in adapting metabolism in response to acidosis [72,73], whereas HIF-1α (i.e., hypoxia-induced transcription factor) shows down-regulated activity [74,75]. As observed in tumor cells of the cervix, colon as well as the pharynx, the enhanced glutamine metabolism occurs as a result of the upregulated activity of HIF-2α as it gets activated under low pH conditions. This glutamine metabolism is regulated by the enhanced expression of the transporter of glutamine known as ASC-like sodium-dependent neutral amino acid transporter 2 (also referred as ASCT2, SLC1A5 and ATB (0)) or GLS1 (referred as glutaminase) instead of the standard metabolism of glucose taking place at neutral pH [76].

Another alteration observed in tumor cell metabolism under low pHe is associated with the metabolism of lipids. The production of reactive oxygen species is also observed in the cells, which are exposed to acute/prolonged time to low pH. However, several phenotypic variations have been observed as tumor cells are chronically in contact with low pH (acidic), causing proliferation (same as that of observed in cancer cells at neutral pH). However, acute exposure to low pH is linked with depicting inhibitions on growth and a rapid reduction in the step where ribose, is obtained from glucose, to be used in the process of ribonucleotide synthesis [77]. Moreover, glutamine and fatty acids contribute to the synthesis of ribose under acute low pH conditions [78]. Such events occur during the progression of cancer cells adapting to low pH conditions and strategies developed for dealing with the increasing demand for survival needs such as bio-energy and antioxidant and afterward developing the capacity to fulfill the biosynthetic needs (lipids and proteins, etc.) to proliferate. The lactate secretion supports cancer cells such as to promote the invasion of the tumor by causing local acidification of the tumor microenvironment (TME), by increasing the protease levels/activities [41,79,80,81,82] or when lactate uptake is carried out by stromal cells to use as a substrate to generate energy for supporting growth and production of pyruvate to be taken up by the cancer cells [83].

### Carbonic Anhydrase Enzyme

The internal cell environment is subjected to various changes due to different extracellular conditions. Therefore, different cellular mechanisms ensure maintaining the homeostatic balance in the internal environment of the cell. One of the mechanisms that play a vital role in maintaining the cellular homeostatic balance is pH, which is partially buffered by a group of membrane-associated enzymes named Carbonic anhydrases (CAs). Carbonic anhydrases are zinc metalloenzymes responsible for reversibly hydrating CO_2_ to yield a bicarbonate molecule (HCO_3_^−^) and a proton (H^+^). At least 15 isoforms of carbonic anhydrase have been identified in mammalian cells with different tissue distribution [84]. CAs exist both intracellularly and extracellularly and were shown to be involved in pH buffering-unrelated functions such as calcium metabolism, signal transduction, metabolism, fluid and electrolyte secretion, and tumorigenesis [85,86,87,88,89].

The tumor microenvironment has a crucial role in tumor invasion and metastasis. The extracellular pH was found to be linked with more invasive phenotypes and aberrant growth of the tumor [90]. One of the mechanisms that allow cancer to resist the low pH is upregulating carbonic anhydrase [90,91]. Reduced invasion of breast cancer cells was observed upon knocking down carbonic anhydrase XII; this was attributed to the concomitant reduction in MMP-2 and u-PA expression levels [92]. CAIX depicted a negative effect on cell adhesion and promote cell motility via augmenting Rho-GTPase signaling cascade [92]. On the other hand, CAIX expression in both normoxic and hypoxic conditions resulted in mediating survival and growth signals [93]. In contrast to the general role of CAs in promoting tumor development and progression, CAII was reported to reverse the epithelial-mesenchymal transition in hepatocellular carcinoma [94]. However, this function may help metastatic cells to seed the destinated tissues by returning the cells to their original morphology.

The state of hypoxia and acidity of the tumor microenvironment represents a challenge for chemotherapeutic agents [95,96,97,98,99,100]. Carbonic anhydrase inhibitors were found to prohibit cancer metastasis; this was mainly linked to increasing the sensitivity of the cancer cells to the anti-angiogenic agents [98]. It was also shown that treating cells with CAIX and CAXII inhibitors promote the susceptibility to radiotherapy-induced apoptosis [97]. Intriguingly, mTOR inhibitor efficacy was stronger in non-hypoxic conditions; nevertheless, the combination of the mTOR inhibitors with CAIX inhibitors portrayed anti-cancer activity under hypoxic conditions [99]. Previous reports revealed that carbonic anhydrases, particularly the membrane-associated isoforms, are considered potential prognostic markers. CAIX demonstrated high levels in the serum of patients with advanced hepatocellular carcinoma [100].

Cancer evolution in response to chemotherapy is becoming a challenge to find an effective chemotherapeutic agent with few side effects. Modulating the pH of the tumor microenvironment by carbonic anhydrase inhibitors has proved to sensitize the tumors to some of the chemotherapeutic agents that show efficacy with fewer side effects.

There are many proteins that regulate bicarbonate dynamics involved in carcinogenesis and becomes more attractive in cancer management (Table 1).

## 6. pH-Tumor Angiogenesis interactions

Tumor vasculature is one of the tumor-microenvironmental hallmarks that play a crucial role in carcinogenesis [102]. Acidic pHe represents a supportive medium for the growth of tumor vessels. To compensate for low extracellular nutrients, tumor cells over-express VEGF and EGF that support the growth of tumor vessels, i.e., VEGF is an adaptive strategy to compensate for tumor acidity and hypoxia [103]. Therefore, in those two opposing factors, tumors develop abnormal vasculatures characterized by the formation of vessels composed of endothelial cells incorporated with transdifferentiated malignant cells resembling endothelial cells (vasculogenic mimicry), which results in a “tumor vasculature mosaicism” [104,105,106].

VEGF, which was originally known as vascular permeability factor (VPF), is a driving force for the neovascularization process. Its expression is activated by acidic pH [107] and most likely results in leaky blood vessels [108,109], which results in less oxygenation, more glycolytic metabolism, and more acidic pH and even more expression of VEGF in the strong positive feedback control loop. Tumor oxygenation may lead to activation of the Krebs’ cycle and so reduces the formation of tumor acidity interstitially and cuts this VEGF cycle.

Bevacizumab is a monoclonal antibody that acts as a VEGF antagonist [110,111]. It will be very attractive due to its likely effect on inhibition/prevention of metastasis, but we are not sure if it targets the endothelial cells alone or endothelial plus tumor cells in the tumor cord. Therefore, a following possible scenario is: If it targets the endothelial cells alone, it may lead to the formation of tumor cord consisting only of tumor cells (population shifting of tumor blood vessels); If it targets both, this diminishes the presence of tumor blood vessels, but it induces selective alterations in the population structure of the tumor neoplasm by putting selective pressure on oxygenated phenotypes, i.e., remove the oxygenated tumor cells and keep the hypoxic and anoxic cells and so provide these later adaptive cells more space. However, those cells are highly responsible for developing metastases [8,39,42].

## 7. Electrolytes-pH Dynamics interactions

Although the above scenario generated the idea that the unique inversion of the pH gradient of cancer cells is a consequence of the perturbation of O_2_ supply, yet another experiment demonstrated that this intracellular pH (pHi) alteration may be the first event of carcinogenesis and that this was independent of O_2_ supply [112]. Whether or not the Warburg effect occurs due to perturbations of O_2_ supply and/or over-expression of NHE1 with a secondary elevation of pHi, there is an increased intracellular pH (intracellular alkalinity) inside the cell which represents suitable intracellular conditions for upregulation of the glycolytic pathway by increasing the expression of several proton exchangers and ion channels, e.g., CLIC1, NaV, Kv, etc.

The ions transport proteins found in the plasma membrane strictly regulate the levels of intracellular pH (i.e., pHi) slightly above neutral inside a normal cell [113]. Several factors influence the regulation of such transporters such as a homeostatic mechanism caused by the intracellular pH change or oncogenes, which are considered as intracellular/extracellular cues [22,114], also consisting of growth factor signaling (83–86), the burden of metabolism (87,88), hypoxia [114] as well as osmotic regulation [115]. However, it has been demonstrated that pHi tends to increase in cancer cells (approx. ∼7.3–7.6) in comparison with the healthy cells (∼7.2), whereas the opposite is found in the extracellular pH (i.e., pHe), which tends to decrease in cancer cells (∼6.8–7.0) as compared to normal cells (∼7.4). This reversed pH gradient is observed in all cancerous cells and suggested that it is an early event of neoplastic development [112] and is found to increase with progression [116]. In cancer cells, the metabolic acids develop due to the increased level of proliferation and metabolism; hence, the increasing level of pHi was early on considered as paradoxical. Although, a high pHi level is kept at a constant level in cancer cells by the plasma membrane ion transporters and the pHi regulators such as the sodium and hydrogen exchanger 1 (also known as NHE1) [114,117], carbonic anhydrases (also referred to as CAs) [118,119], monocarboxylate transporter 1 and 4 (i.e., MCT1 and 4) [115], as well as a sodium-driven bicarbonate exchanger [120,121,122].

The pH dysregulation observed in cancer cells helps to drive tumor processes, which are highly sensitive to even minimum pHi variations: these are cancer initiation, progression, adaptation, proliferation, migration, and the programming and reprogramming of tumor cell metabolism [14,123,124,125,126,127]. Furthermore, many effects on biologic processes of a cell are generated as a result of pH-sensitive functions such as the regulation of protein expression, the affinity of proteins to bind with a ligand as well as the activity of ion channels/enzymes [124,128]. Some of these pH-sensitive ion channels, enzymes [124,129,130], and processes are described below.

### 7.1. Sodium-Ion (Na^+^)

Although cancer cells are usually considered as non-excitable cells, they do express Voltage-gated sodium channels (NaV) in a relatively abundant amount [131] that opposes the prevailing dogma of a role only in excitable tissue physiology [131]. As a non-excitable protein, NaV supports tumor growth [132,133], invasion [134,135], metastasis [135,136] and associated with prognosis [134] and correlated with resistance to treatment [131]. Therefore, it represents an attractive target for therapy [137,138].

Tetrodotoxin (TTX), aminoperhydroquinazoline, is a poisonous alkaloidal compound found mainly in the liver and ovaries of fishes in the order Tetraodontiformes. This compound causes paresthesia and paralysis via alteration with neuromuscular conduction [139,140]. TTX is a well-known NaV-Inhibitor [141]. It is a neurotoxin used analgesic in relieving cancer pain (palliative treatment) [142,143,144]. TTX blocks NaV extracellularly, while local anesthesia blocks NaV intracellularly with the various anticonvulsant drug, e.g., Phenytoin, which is considered a potential anticancer agent [145]. The class 1C antiarrhythmic agent, propafenone, also can act as an anticancer [146].

Many proteins regulate sodium dynamics involved in carcinogenesis and become more attractive in cancer management (Table 2).

### 7.2. Potassium Ion (K^+^)

In an early report, the ratio of [K^+^]_i_/[Na^+^]_i_ inside the cell was decreased in both aging and cancer development [156], while other data showed a higher level of potassium. This contradictory data reveals that potassium may be more elevated extracellularly and reduced intracellularly in cancer. This is supported by other data that demonstrate that extracellular potassium suppresses the immune system [157]. Moreover, much pharmacological evidence supports the current hypothesis because Amiloride and Cariporide (potassium-sparing diuretics) and well known as NHE1 inhibitors are considered to be potential anticancer agents [1,151,152,158]. Therefore, it will not be surprising if potassium ion channels are correlated with malignant transformation via outward K^+^ extracellular flow [159,160].

One of these channels is a voltage-gated potassium channel (VGKC) highly specific for potassium (filter selectivity at the extracellular domain) and sensitive to voltage changes in the cell’s membrane potential. VCKCs are most active at an acidic pH and are associated with various diseases, including cancer [161]. VGKCs play a crucial role in cellular proliferation [162], potential metastasis [163], and drug resistance [164].

The non-sedating antihistamine, Astemizole, has been withdrawn from the market due to its prolonging of the QT interval. However, due to this property, it could act as a potential agent against VGKCs (e.g., off-label use of it as anti-cancer) [165].

Many proteins regulate potassium dynamics involved in carcinogenesis and become more attractive in cancer management (Table 3).

### 7.3. Calcium Ion (Ca^2+^)

While the calcium ion (Ca^2+^) acts as a second messenger, it also has many essential roles in the cells, including gene expression, cycle, cellular motility, besides its role in apoptosis [173,174,175].

The exact role of calcium in cancer is a conundrum because, on the one hand, releasing calcium is accompanied by activating cytochrome C and leads to a downstream cascade of apoptosis, including apoptosome and other apoptotic intermediates involved in the process of the programmed cell death [176,177]. On the other hand, calcium supports the cell cycle [178,179,180,181] and cellular migration, too [182,183,184]. Therefore, some calcium channels, e.g., Voltage-gated calcium (Ca^2+^) channels (VGCC), increased in tumors [185,186,187,188,189] while other types of channels were decreased in other tumors (e.g., Ca^2+^- ATPase (SERCA3, PMCA1, PMCA4)) [190,191,192]. Moreover, even the same channel may fluctuate among different tumors, e.g., TRPV1 is increased in prostate cancer [193] and decreased in Bladder cancer [194]. Thus, it may be too early to reach a conclusion that draws a clear demarcation in answering when and where the calcium supports the tumorigenesis or blocking it. However, the possible explanation may come through studying the kinetics and subcellular localization of Ca^2+^ signals essential for the identification of which Ca^2+^-dependent cascade(s) are stimulated and in the duration of action.

Such a challenging scenario is extended to use the calcium channel regulators/modulators (inhibitors and/or agonist) as therapeutics in cancer, e.g., cannabidiol (CBD) (a derivative of CBDA) and capsaicin are agonists of TRPV1 and used to treat several types of cancer including colon and renal cancer [173]. Carboxyamidotriazole and dihydropyridine inhibit Orai (CRAC) and used in the treatment of several tumors, e.g., hepatoma, lung, and glioma [173]. Also, Verapamil as VGCC-blocker and potassium channel blocker can be used as chemosensitizers [195].

Many proteins regulate calcium ion dynamics involved in carcinogenesis and are becoming more attractive in cancer management (Table 4).

### 7.4. Chloride Ion (Cl^−^)

Chloride (Cl^−^) channels are paramount in the physiology of the body include contraction of the muscle, neuronal excitability, cell-volume osmoregulation, transepithelial fluid transportation, production, and secretion of mucus and intracellular organelles acidification [197,198]. One of those channels is Chloride intracellular channel protein 1 (CLIC1).

CLIC1, also known as G6, NCC27, or CLCNL1, is an abundant protein that can be found in both soluble, an unusual form of the ion channel, as well as a nuclear membrane (e.g., nucleoplasm) associated form [199,200,201]. This protein is related to chloride ion transport in various cellular compartments [202] and is also found in cytoplasmic organelles, e.g., lysosomes, endosomes, and secretory vesicles [201]. CLIC1 plays a role in the redox state of the cell by modulating Reactive Oxygen Species (ROS) [200], and both its function and incorporation into the membrane and its function are governed by both the redox state of the cell [203] and pH [199]. Further, both acidic pHe [199] and H_2_O_2_ [204] support the formation of a membrane-associated form. This may reveal its importance in the manipulation of ROS in attacking the nucleus. Therefore, some have found CLIC1 challenging to consider it as an ion channel or not [200]. Due to its role in regulating ROS, the CLIC1 ion channels are preferentially expressed in cancer cells with an oxidative phenotype that cluster around blood vessels (i.e., relatively abundant in oxygen supply) [8,39]. Overexpression of this protein is correlated with tumor growth [205,206], invasion, and metastasis [207,208,209,210] and poor prognosis [211,212,213], while its role in chemotherapy is controversial [214,215]. It has been discovered that CLIC1 maintains the population size of the neoplasm [216].

R (+) Indanyloxyacetic acid 94 (R(+)-IAA-94) inhibits the CLIC1 ion channel [200,217] as does Metformin [218]. Its inhibition by metformin could indicate a possible correlation of CLIC1 with the cellular metabolic state.

Many proteins regulate chloride ion dynamics involved in carcinogenesis and are becoming more attractive in cancer management (Table 5).

## 8. Conclusions

It is now well-accepted that one of the unifying characteristics of cancer is its strong pH dysregulation and reversal of the normal pHi to pHe gradient. Various studies have suggested that this pH dysregulation drives most if not all of the tumor hallmarks and the resistance to many chemotherapeutic agents. This reversed pH gradient can be reduced or even ablated through suppression of the overall expression and/or activity of the ion transporters and enzymes that drive it. Indeed, there is now ample evidence that specific inhibitors of ion transporters (Carbonic anhydrase, sodium-hydrogen exchangers, Bicarbonate transporters, etc.), is enough to re-sensitize a resistant cell line to chemotherapy. Importantly, since most of these transporters are quiescent in normal tissues and are usually activated to maintain the cells in the slightly above neutral pHi during systemic acidosis &/or alkalosis, while these transporters or enzymes are over activated in the cancer cells reduces the possibility of side effects occurring during treatment [152]. Therefore, to target the intracellular alkaline pHi of cancer cells as adjuvant therapy with several other chemotherapeutic drugs holds promise as a practical approach. Importantly, Cancer also has another crucial dimension in that it can be seen as a disease of unusual electrolyte levels. For instance, increasing the sodium level supports tumorigenesis, while a higher level of potassium interrupts it. Also, chloride, calcium, and bicarbonate ions have long been associated with tumorigenesis. In this respect, many inhibitors of proton transporters and ion channels that influence cellular pH also influence cellular electrolyte levels and dynamics.

Moreover, the acidic extracellular pHe causes attenuation of the immune response, and neutralizing pHe is effective in improving the responses towards the cancer immunity-based therapies. Many studies in cell lines, tumors, and animal models have shown promising results for inhibiting ion transporters and have suggested readily clinical applications. However, future studies may help in revealing new combined therapies that make use of treatments specific for genetic signature as well as histological identification along with essential inhibitors of ion transporters.

## Figures and Tables

**Table 1:** Proteins that regulate bicarbonate dynamics involved in carcinogenesis with its potential modulators.

The Name of Proteins	Modulators
Anion exchanger family (bicarbonate transporter family)	Acetazolamide, niflumic acid [101]

**Table 2:** Proteins that regulate sodium ion dynamics involved in carcinogenesis with potential modulators.

The Name of Proteins	Modulators
Voltage-gated sodium channels (NaV)	Tetrodotoxin (TTX)
Sodium-calcium exchanger (Na^+^/Ca^2+^ exchangers; NCX)	Bepridil, 3′,4′-dichlorobenzamil hydrochloride, KB-R7943, SEA0400, SN-6, YM-244769 [147,148,149,150]
Sodium–hydrogen antiporter or sodium–proton exchanger (Na^+^/H^+^ exchanger; NHE)	cariporide, amiloride; HMA (5-(N,N-hexametylene)-amiloride); Phx-3; Compound 9t [1,116,123,128,151,152]
Na^+^/K^+^-ATPase (sodium - potassium adenosine triphosphatase; the Na^+^/K^+^ pump or sodium–potassium pump)	Digoxin; Ouabain; 3,4,5,6-tetrahydroxyxanthone [153,154,155]
Potassium-dependent sodium-calcium exchanger	-

**Table 3:** Proteins that regulate potassium ion dynamics involved in carcinogenesis with potential modulators.

The Name of Proteins		Modulators
Voltage-gated potassium channel (VGKCs)		Astemizole
Calcium-activated potassium channel	BK channel (Maxi-K, slo1)	Charybdotoxin (blocker) [166]
	SK channels	Rottlerin (mallotoxin) (activator) [167]
	IK channel	
Inwardly rectifying potassium channel	The renal outer medullary potassium channel (ROMK)	Nicorandil (could be used as adjuvant therapy to anticancer to prevent cardiotoxicity) [168,169]
	G protein-coupled inwardly rectifying potassium channels (GIRKs)	
	ATP-sensitive potassium channel (KATP channel)	
Tandem pore domain potassium channel		Fluoxetine [170,171,172]

**Table 4:** Proteins that regulate calcium ion dynamics involved in carcinogenesis with potential modulators.

The Name of Proteins		Modulators
voltage-gated calcium channel	L-type calcium channel	Mibefradil [196]
	P-type calcium channel/Q-type calcium channel	
	N-type calcium channel	
	R-type calcium channel	
	T-type calcium channel	
ligand-gated calcium channel	IP3 receptor (Inositol trisphosphate receptor (InsP3R))	Carboxyamidotriazole and dihydropyridine
	Ryanodine receptor	
	Two-pore channel	
	cation channels of sperm; Catsper channels (CatSper)	
	store-operated channels	

**Table 5:** Proteins that regulate chloride ion dynamics involved in carcinogenesis with potential modulators.

The Name of Proteins	Modulators
CLC family	5-nitro-2-3-phenylpropylamino benzoic acid (NPPB), [219]
Epithelial Chloride Channel (E-ClC) family	sodium butyrate [220]
Chloride Intracellular Ion Channel (CLIC) Family	R (+) Indanyloxyacetic acid 94 (R(+)-IAA-94)
Cystic fibrosis transmembrane conductance regulator (CFTR)	-

## References

[1] Alfarouk K.O., Verduzco D., Rauch C., Muddathir A.K., Bashir A.H.H., Elhassan G.O., Ibrahim M.E., Orozco P.J.D., Cardone R.A., Reshkin S.J. (2014). Glycolysis, tumor metabolism, cancer growth and dissemination. A new pH-based etiopathogenic perspective and therapeutic approach to an old cancer question. Oncoscience.

[2] Alfarouk K.O. (2016). Tumor metabolism, cancer cell transporters, and microenvironmental resistance. J. Enzyme Inhib. Med. Chem..

[3] Juel C., Halestrap A.P. (1999). Lactate transport in skeletal muscle—Role and regulation of the monocarboxylate transporter. J. Physiol..

[4] Gladden L.B. (2011). Lactate Transport and Exchange During Exercise. Comprehensive Physiology.

[5] Korenchan D.E., Flavell R.R. (2019). Spatiotemporal pH Heterogeneity as a Promoter of Cancer Progression and Therapeutic Resistance. Cancers.

[6] Swietach P. (2019). What is pH regulation, and why do cancer cells need it?. Cancer Metastasis Rev..

[7] McDonald P.C., Chafe S.C., Brown W.S., Saberi S., Swayampakula M., Venkateswaran G., Nemirovsky O., Gillespie J.A., Karasinska J.M., Kalloger S.E. (2019). Regulation of pH by Carbonic Anhydrase 9 Mediates Survival of Pancreatic Cancer Cells With Activated KRAS in Response to Hypoxia. Gastroenterology.

[8] Alfarouk K.O., Shayoub M.E.A., Muddathir A.K., Elhassan O., Bashir A.H.H. (2011). Evolution of tumor metabolism might reflect carcinogenesis as a reverse evolution process (dismantling of multicellularity). Cancers.

[9] Duncker D.J., Bache R.J. (2008). Regulation of Coronary Blood Flow During Exercise. Physiol. Rev..

[10] Laughlin M.H., Roseguini B. (2008). Mechanisms for exercise training-induced increases in skeletal muscle blood flow capacity: Differences with interval sprint training versus aerobic endurance training. J. Physiol. Pharmacol..

[11] Laughlin M.H. (2016). Physical activity-induced remodeling of vasculature in skeletal muscle: Role in treatment of type 2 diabetes. J. Appl. Physiol..

[12] Certo M., Del Gaizo Moore V., Nishino M., Wei G., Korsmeyer S., Armstrong S.A., Letai A. (2006). Mitochondria primed by death signals determine cellular addiction to antiapoptotic BCL-2 family members. Cancer Cell.

[13] Hoppeler H., Vogt M., Weibel E.R., Flück M. (2003). Response of Skeletal Muscle Mitochondria to Hypoxia. Exp. Physiol..

[14] Schwartz L., Supuran C.T., Alfarouk K.O. (2017). The Warburg effect and the Hallmarks of Cancer. Anticancer. Agents Med. Chem..

[15] Tapia L., Pérez Y., Bolte M., Casas J., Solà J., Quesada R., Alfonso I. (2019). pH-Dependent Chloride Transport by Pseudopeptidic Cages for the Selective Killing of Cancer Cells in Acidic Microenvironments. Angew. Chemie Int. Ed..

[16] Glitsch M. (2011). Protons and Ca^2+^: Ionic allies in tumor progression?. Physiology.

[17] Justus C.R., Dong L., Yang L.V. (2013). Acidic tumor microenvironment and pH-sensing G protein-coupled receptors. Front. Physiol..

[18] Damaghi M., Wojtkowiak J.W., Gillies R.J. (2013). pH sensing and regulation in cancer. Front. Physiol..

[19] Maly I.V., Hofmann W.A. (2018). Calcium and nuclear signaling in prostate cancer. Int. J. Mol. Sci..

[20] Albensi B.C. (2019). What Is Nuclear Factor Kappa B (NF-κB) Doing in and to the Mitochondrion?. Front. Cell Dev. Biol..

[21] Pouysségur J., Sardet C., Franchi A., L’Allemain G., Paris S. (1984). A specific mutation abolishing Na^+^/H^+^ antiport activity in hamster fibroblasts precludes growth at neutral and acidic pH. Proc. Natl. Acad. Sci. USA.

[22] Grillo-Hill B.K., Choi C., Jimenez-Vidal M., Barber D.L. (2015). Increased H^+^ efflux is sufficient to induce dysplasia and necessary for viability with oncogene expression. eLife.

[23] Lauritzen G., Stock C.-M., Lemaire J., Lund S.F., Jensen M.F., Damsgaard B., Petersen K.S., Wiwel M., Rønnov-Jessen L., Schwab A. (2012). The Na^+^/H^+^ exchanger NHE1, but not the Na^+^, cotransporter NBCn1, regulates motility of MCF7 breast cancer cells expressing constitutively active ErbB2. Cancer Lett..

[24] Boedtkjer E., Moreira J.M.A., Mele M., Vahl P., Wielenga V.T., Christiansen P.M., Jensen V.E.D., Pedersen S.F., Aalkjaer C. (2013). Contribution of Na^+^,HCO_3_^−^-cotransport to cellular pH control in human breast cancer: A role for the breast cancer susceptibility locus NBCn1 (SLC4A7). Int. J. Cancer.

[25] McIntyre A., Hulikova A., Ledaki I., Snell C., Singleton D., Steers G., Seden P., Jones D., Bridges E., Wigfield S. (2016). Disrupting Hypoxia-Induced Bicarbonate Transport Acidifies Tumor Cells and Suppresses Tumor Growth. Cancer Res..

[26] Goh W., Sleptsova-Freidrich I., Petrovic N. (2014). Use of proton pump inhibitors as adjunct treatment for triple-negative breast cancers. An introductory study. J. Pharm. Pharm. Sci..

[27] Slepkov E., Fliegel L. (2002). Structure and function of the NHE1 isoform of the Na^+^/H^+^ exchanger. Biochem. Cell Biol..

[28] Gottlieb R.A., Nordberg J., Skowronski E., Babior B.M. (1996). Apoptosis induced in Jurkat cells by several agents is preceded by intracellular acidification. Proc. Natl. Acad. Sci. USA.

[29] Matsuyama S., Llopis J., Deveraux Q.L., Tsien R.Y., Reed J.C. (2000). Changes in intramitochondrial and cytosolic pH: Early events that modulate caspase activation during apoptosis. Nat. Cell Biol..

[30] Lagadic-Gossmann D., Huc L., Lecureur V. (2004). Alterations of intracellular pH homeostasis in apoptosis: Origins and roles. Cell Death Differ..

[31] Liu D., Martino G., Thangaraju M., Sharma M., Halwani F., Shen S.H., Patel Y.C., Srikant C.B. (2000). Caspase-8-mediated intracellular acidification precedes mitochondrial dysfunction in somatostatin-induced apoptosis. J. Biol. Chem..

[32] Mansouri A., Ridgway L.D., Zhang Q., Claret F.X. (2001). Activation of the JNK/SAPK and P38 Mitogen-Activated Protein Kinase Signaling Pathways Sensitize Tumor Cells to Cisplatin-Induced Apoptosis. Sci. World J..

[33] Pérez-Sala D., Collado-Escobar D., Mollinedo F. (1995). Intracellular alkalinization suppresses lovastatin-induced apoptosis in HL-60 cells through the inactivation of a pH-dependent endonuclease. J. Biol. Chem..

[34] Sonveaux P., Végran F., Schroeder T., Wergin M.C., Verrax J., Rabbani Z.N., De Saedeleer C.J., Kennedy K.M., Diepart C., Jordan B.F. (2008). Targeting lactate-fueled respiration selectively kills hypoxic tumor cells in mice. J. Clin. Invest..

[35] Nakajima E.C., Van Houten B. (2013). Metabolic symbiosis in cancer: Refocusing the Warburg lens. Mol. Carcinog..

[36] Jose C., Bellance N., Rossignol R. (2011). Choosing between glycolysis and oxidative phosphorylation: A tumor’s dilemma?. Biochim. Biophys. Acta Bioenerg..

[37] Smolková K., Plecitá-Hlavatá L., Bellance N., Benard G., Rossignol R., Ježek P. (2011). Waves of gene regulation suppress and then restore oxidative phosphorylation in cancer cells. Int. J. Biochem. Cell Biol..

[38] Pavlides S., Whitaker-Menezes D., Castello-Cros R., Flomenberg N., Witkiewicz A.K., Frank P.G., Casimiro M.C., Wang C., Fortina P., Addya S. (2009). The reverse Warburg effect: Aerobic glycolysis in cancer associated fibroblasts and the tumor stroma. Cell Cycle.

[39] Alfarouk K.O., Ibrahim M.E., Gatenby R.A., Brown J.S. (2013). Riparian ecosystems in human cancers. Evol. Appl..

[40] Lloyd M.C., Alfarouk K.O., Verduzco D., Bui M.M., Gillies R.J., Ibrahim M.E., Brown J.S., Gatenby R.A. (2014). Vascular measurements correlate with estrogen receptor status. BMC Cancer.

[41] Alfarouk K.O., Muddathir A.K., Shayoub M.E.A. (2011). Tumor acidity as evolutionary spite. Cancers.

[42] Alfarouk K.O., Stock C.-M., Taylor S., Walsh M., Muddathir A.K., Verduzco D., Bashir A.H., Mohammed O.Y., Elhassan G.O., Harguindey S. (2015). Resistance to cancer chemotherapy: Failure in drug response from ADME to P-gp. Cancer Cell Int..

[43] Fantin V.R., St-Pierre J., Leder P. (2006). Attenuation of LDH-A expression uncovers a link between glycolysis, mitochondrial physiology, and tumor maintenance. Cancer Cell.

[44] Xie H., Hanai J., Ren J.-G., Kats L., Burgess K., Bhargava P., Signoretti S., Billiard J., Duffy K.J., Grant A. (2014). Targeting Lactate Dehydrogenase-A Inhibits Tumorigenesis and Tumor Progression in Mouse Models of Lung Cancer and Impacts Tumor-Initiating Cells. Cell Metab..

[45] Zhao D., Zou S.-W., Liu Y., Zhou X., Mo Y., Wang P., Xu Y.-H., Dong B., Xiong Y., Lei Q.-Y. (2013). Lysine-5 Acetylation Negatively Regulates Lactate Dehydrogenase A and Is Decreased in Pancreatic Cancer. Cancer Cell.

[46] Read J.A., Winter V.J., Eszes C.M., Sessions R.B., Brady R.L. (2001). Structural basis for altered activity of M- and H-isozyme forms of human lactate dehydrogenase. Proteins.

[47] Isom D.G., Sridharan V., Baker R., Clement S.T., Smalley D.M., Dohlman H.G. (2013). Protons as Second Messenger Regulators of G Protein Signaling. Mol. Cell.

[48] Andrés V., Carreras J., Cussó R. (1990). Regulation of muscle phosphofructokinase by physiological concentrations of bisphosphorylated hexoses: Effect of alkalinization. Biochem. Biophys. Res. Commun..

[49] Moreno-Sánchez R., Marín-Hernández A., Gallardo-Pérez J.C., Quezada H., Encalada R., Rodríguez-Enríquez S., Saavedra E. (2012). Phosphofructokinase type 1 kinetics, isoform expression, and gene polymorphisms in cancer cells. J. Cell. Biochem..

[50] Webb B.A., Forouhar F., Szu F.-E., Seetharaman J., Tong L., Barber D.L. (2015). Structures of human phosphofructokinase-1 and atomic basis of cancer-associated mutations. Nature.

[51] Kawada K., Toda K., Sakai Y. (2017). Targeting metabolic reprogramming in KRAS-driven cancers. Int. J. Clin. Oncol..

[52] Karnoub A.E., Weinberg R.A. (2008). Ras oncogenes: Split personalities. Nat. Rev. Mol. Cell Biol..

[53] Mukhopadhyay S., Saqcena M., Foster D.A. (2015). Synthetic lethality in KRas-driven cancer cells created by glutamine deprivation. Oncoscience.

[54] Yun J., Rago C., Cheong I., Pagliarini R., Angenendt P., Rajagopalan H., Schmidt K., Willson J.K.V., Markowitz S., Zhou S. (2009). Glucose Deprivation Contributes to the Development of KRAS Pathway Mutations in Tumor Cells. Science.

[55] Hutton J.E., Wang X., Zimmerman L.J., Slebos R.J.C., Trenary I.A., Young J.D., Li M., Liebler D.C. (2016). Oncogenic KRAS and BRAF Drive Metabolic Reprogramming in Colorectal Cancer. Mol. Cell. Proteom..

[56] Yun J., Mullarky E., Lu C., Bosch K.N., Kavalier A., Rivera K., Roper J., Chio I.I.C., Giannopoulou E.G., Rago C. (2015). Vitamin C selectively kills KRAS and BRAF mutant colorectal cancer cells by targeting GAPDH. Science.

[57] Tai C.-J., Wang W.-C., Wang C.-K., Wu C.-H., Yang M.-D., Chang Y.-J., Jian J.-Y., Tai C.-J. (2013). Fermented Wheat Germ Extract Induced Cell Death and Enhanced Cytotoxicity of Cisplatin and 5-Fluorouracil on Human Hepatocellular Carcinoma Cells. Evidence-Based Complement. Altern. Med. Suppl..

[58] Comín-Anduix B., Boros L.G.G., Marin S., Boren J., Callol-Massot C., Centelles J.J.J., Torres J.L.L., Agell N., Bassilian S., Cascante M. (2002). Fermented Wheat Germ Extract Inhibits Glycolysis/Pentose Cycle Enzymes and Induces Apoptosis through Poly(ADP-ribose) Polymerase Activation in Jurkat T-cell Leukemia Tumor Cells. J. Biol. Chem..

[59] Schwartz L., Seyfried T., Alfarouk K.O.K.O., Da Veiga Moreira J., Fais S. (2017). Out of Warburg effect: An effective cancer treatment targeting the tumor specific metabolism and dysregulated pH. Semin. Cancer Biol..

[60] Spugnini E.P., Fais S. (2020). Drug repurposing for anticancer therapies. A lesson from proton pump inhibitors. Expert Opin. Ther. Pat..

[61] Ding D.C., Sung F.C., Chen W., Wang J.H., Lin S.Z. (2019). Proton pump inhibitors reduce breast cancer risk in gastric ulcer patients: A population-based cohort study. Breast J..

[62] Chen C.H., Lee C.Z., Lin Y.C., Kao L.T., Lin H.C. (2019). Negative Association of Proton Pump Inhibitors With Subsequent Development of Breast Cancer: A Nationwide Population-Based Study. J. Clin. Pharmacol..

[63] Khacho M., Tarabay M., Patten D., Khacho P., MacLaurin J.G., Guadagno J., Bergeron R., Cregan S.P., Harper M.-E., Park D.S. (2014). Acidosis overrides oxygen deprivation to maintain mitochondrial function and cell survival. Nat. Commun..

[64] Corbet C., Draoui N., Polet F., Pinto A., Drozak X., Riant O., Feron O. (2014). The SIRT1/HIF2α Axis Drives Reductive Glutamine Metabolism under Chronic Acidosis and Alters Tumor Response to Therapy. Cancer Res..

[65] Filatova A., Seidel S., Böğürcü N., Gräf S., Garvalov B.K., Acker T. (2016). Acidosis Acts through HSP90 in a PHD/VHL-Independent Manner to Promote HIF Function and Stem Cell Maintenance in Glioma. Cancer Res..

[66] Kondo A., Yamamoto S., Nakaki R., Shimamura T., Hamakubo T., Sakai J., Kodama T., Yoshida T., Aburatani H., Osawa T. (2017). Extracellular Acidic pH Activates the Sterol Regulatory Element-Binding Protein 2 to Promote Tumor Progression. Cell Rep..

[67] Parks S.K., Chiche J., Pouysségur J. (2013). Disrupting proton dynamics and energy metabolism for cancer therapy. Nat. Rev. Cancer.

[68] Parks S.K., Mazure N.M., Counillon L., Pouysségur J. (2013). Hypoxia promotes tumor cell survival in acidic conditions by preserving ATP levels. J. Cell. Physiol..

[69] Boidot R., Vegran F., Meulle A., Le Breton A., Dessy C., Sonveaux P., Lizard-Nacol S., Feron O. (2011). Regulation of Monocarboxylate Transporter MCT1 Expression by p53 Mediates Inward and Outward Lactate Fluxes in Tumors. Cancer Res..

[70] Végran F., Boidot R., Michiels C., Sonveaux P., Feron O., Vegran F., Boidot R., Michiels C., Sonveaux P., Feron O. (2011). Lactate influx through the endothelial cell monocarboxylate transporter MCT1 supports an NF-κB/IL-8 pathway that drives tumor angiogenesis. Cancer Res..

[71] Pisarsky L., Bill R., Fagiani E., Dimeloe S., Goosen R.W., Hagmann J., Hess C., Christofori G. (2016). Targeting Metabolic Symbiosis to Overcome Resistance to Anti-angiogenic Therapy. Cell Rep..

[72] Mekhail K., Gunaratnam L., Bonicalzi M.-E., Lee S. (2004). HIF activation by pH-dependent nucleolar sequestration of VHL. Nat. Cell Biol..

[73] Hjelmeland A.B., Wu Q., Heddleston J.M., Choudhary G.S., MacSwords J., Lathia J.D., McLendon R., Lindner D., Sloan A., Rich J.N. (2011). Acidic stress promotes a glioma stem cell phenotype. Cell Death Differ..

[74] Tang X., Lucas J.E., Chen J.L.-Y., LaMonte G., Wu J., Wang M.C., Koumenis C., Chi J.-T. (2012). Functional Interaction between Responses to Lactic Acidosis and Hypoxia Regulates Genomic Transcriptional Outputs. Cancer Res..

[75] Chen J.L.-Y., Lucas J.E., Schroeder T., Mori S., Wu J., Nevins J., Dewhirst M., West M., Chi J.-T. (2008). The Genomic Analysis of Lactic Acidosis and Acidosis Response in Human Cancers. PLoS Genet..

[76] Dioum E.M., Chen R., Alexander M.S., Zhang Q., Hogg R.T., Gerard R.D., Garcia J.A. (2009). Regulation of Hypoxia-Inducible Factor 2 Signaling by the Stress-Responsive Deacetylase Sirtuin 1. Science.

[77] Schug Z.T., Peck B., Jones D.T., Zhang Q., Grosskurth S., Alam I.S., Goodwin L.M., Smethurst E., Mason S., Blyth K. (2015). Acetyl-CoA synthetase 2 promotes acetate utilization and maintains cancer cell growth under metabolic stress. Cancer Cell.

[78] LaMonte G., Tang X., Ling-Yu Chen J., Wu J., Cornelia Ding C.-K., Keenan M.M., Sangokoya C., Kung H.-N., Ilkayeva O., Boros L.G. (2013). Acidosis induces reprogramming of cellular metabolism to mitigate oxidative stress Cancer & Metabolism. Cancer Metab..

[79] Schoch H.J., Fischer S., Marti H.H. (2002). Hypoxia-induced vascular endothelial growth factor expression causes vascular leakage in the brain. Brain.

[80] Shi Q., Le X., Wang B., Abbruzzese J.L., Xiong Q., He Y., Xie K. (2001). Regulation of vascular endothelial growth factor expression by acidosis in human cancer cells. Oncogene.

[81] Baumann F., Leukel P., Doerfelt A., Beier C.P., Dettmer K., Oefner P.J., Kastenberger M., Kreutz M., Nickl-Jockschat T., Bogdahn U. (2009). Lactate promotes glioma migration by TGF-beta2-dependent regulation of matrix metalloproteinase-2. Neuro. Oncol..

[82] Rothberg J.M., Bogyo M.S., Sloane B.F. (2012). Abstract 2467: Acidic pericellular pH increases contribution of cathepsin B to invasiveness of a human breast carcinoma cell line. Cancer Res..

[83] Hirschhaeuser F., Sattler U.G.A., Mueller-Klieser W. (2011). Lactate: A metabolic key player in cancer. Cancer Res..

[84] Singh S., Lomelino C., Mboge M., Frost S., McKenna R. (2018). Cancer Drug Development of Carbonic Anhydrase Inhibitors beyond the Active Site. Molecules.

[85] Supuran C.T. (2018). Carbonic Anhydrases and Metabolism. Metabolites.

[86] Gram J., Bollerslev J., Nielsen H.K., Larsen H.F., Mosekilde L. (1990). The effect of carbonic anhydrase inhibition on calcium and bone homeostasis in healthy postmenopausal women. J. Intern. Med..

[87] Hong J.H., Muhammad E., Zheng C., Hershkovitz E., Alkrinawi S., Loewenthal N., Parvari R., Muallem S. (2015). Essential role of carbonic anhydrase XII in secretory gland fluid and HCO_3_− secretion revealed by disease causing human mutation. J. Physiol..

[88] Dorai T., Sawczuk I.S., Pastorek J., Wiernik P.H., Dutcher J.P. (2005). The role of carbonic anhydrase IX overexpression in kidney cancer. Eur. J. Cancer.

[89] Eom K.-Y., Jang M.H., Park S.Y., Kang E.Y., Kim S.W., Kim J.H., Kim J.-S., Kim I.A. (2016). The Expression of Carbonic Anhydrase (CA) IX/XII and Lymph Node Metastasis in Early Breast Cancer. Cancer Res. Treat..

[90] Mboge M.Y., Mahon B.P., McKenna R., Frost S.C. (2018). Carbonic Anhydrases: Role in pH Control and Cancer. Metabolites.

[91] Logozzi M., Capasso C., Di Raimo R., Del Prete S., Mizzoni D., Falchi M., Supuran C.T., Fais S. (2019). Prostate cancer cells and exosomes in acidic condition show increased carbonic anhydrase IX expression and activity. J. Enzyme Inhib. Med. Chem..

[92] Hsieh M.-J., Chen K.-S., Chiou H.-L., Hsieh Y.-S. (2010). Carbonic anhydrase XII promotes invasion and migration ability of MDA-MB-231 breast cancer cells through the p38 MAPK signaling pathway. Eur. J. Cell Biol..

[93] Jang B.G., Yun S.-M., Ahn K., Song J.H., Jo S.A., Kim Y.-Y., Kim D.K., Park M.H., Han C., Koh Y.H. (2010). Plasma Carbonic Anhydrase II protein is Elevated in Alzheimer’s Disease. J. Alzheimer’s Dis..

[94] Zhang C., Wang H., Chen Z., Zhuang L., Xu L., Ning Z., Zhu Z., Wang P., Meng Z. (2018). Carbonic anhydrase 2 inhibits epithelial–mesenchymal transition and metastasis in hepatocellular carcinoma. Carcinogenesis.

[95] Cosse J.-P., Michiels C. (2008). Tumour hypoxia affects the responsiveness of cancer cells to chemotherapy and promotes cancer progression. Anticancer. Agents Med. Chem..

[96] Wojtkowiak J.W., Verduzco D., Schramm K.J., Gillies R.J. (2011). Drug resistance and cellular adaptation to tumor acidic pH microenvironment. Mol. Pharm..

[97] Ward C., Meehan J., Gray M., Kunkler I., Langdon S., Argyle D. (2018). Carbonic Anhydrase IX (CAIX), Cancer, and Radiation Responsiveness. Metabolites.

[98] Vaeteewoottacharn K., Kariya R., Dana P., Fujikawa S., Matsuda K., Ohkuma K., Kudo E., Kraiklang R., Wongkham C., Wongkham S. (2016). Inhibition of carbonic anhydrase potentiates bevacizumab treatment in cholangiocarcinoma. Tumor Biol..

[99] Faes S., Planche A., Uldry E., Santoro T., Pythoud C., Stehle J.-C., Horlbeck J., Letovanec I., Riggi N., Datta D. (2016). Targeting carbonic anhydrase IX improves the anti-cancer efficacy of mTOR inhibitors. Oncotarget.

[100] Kang H.J., Kim I.H., Sung C.O., Shim J.H., Yu E. (2015). Expression of carbonic anhydrase 9 is a novel prognostic marker in resectable hepatocellular carcinoma. Virchows Arch..

[101] Čeponytė U., Paškevičiūtė M., Petrikaitė V. (2018). Comparison of NSAIDs activity in COX-2 expressing and non-expressing 2D and 3D pancreatic cancer cell cultures. Cancer Manag. Res..

[102] Hanahan D., Weinberg R.A. (2011). Hallmarks of cancer: The next generation. Cell.

[103] Lin C., McGough R., Aswad B., Block J.A., Terek R. (2004). Hypoxia induces HIF-1α and VEGF expression in chondrosarcoma cells and chondrocytes. J. Orthop. Res..

[104] Chang Y.S., di Tomaso E., McDonald D.M., Jones R., Jain R.K., Munn L.L. (2000). Mosaic blood vessels in tumors: Frequency of cancer cells in contact with flowing blood. Proc. Natl. Acad. Sci. USA.

[105] Folkman J. (2001). Can mosaic tumor vessels facilitate molecular diagnosis of cancer?. Proc. Natl. Acad. Sci. USA.

[106] Di Tomaso E., Capen D., Haskell A., Hart J., Logie J.J., Jain R.K., McDonald D.M., Jones R., Munn L.L. (2005). Mosaic tumor vessels: Cellular basis and ultrastructure of focal regions lacking endothelial cell markers. Cancer Res..

[107] Xu L., Fukumura D., Jain R.K. (2002). Acidic extracellular pH induces vascular endothelial growth factor (VEGF) in human glioblastoma cells via ERK1/2 MAPK signaling pathway: Mechanism of low pH-induced VEGF. J. Biol. Chem..

[108] McDonald D.M., Baluk P. (2002). Significance of blood vessel leakiness in cancer. Cancer Res..

[109] Nagy J.A., Chang S.-H., Dvorak A.M., Dvorak H.F. (2009). Why are tumour blood vessels abnormal and why is it important to know?. Br. J. Cancer.

[110] Ferrara N., Hillan K.J., Gerber H.-P., Novotny W. (2004). Discovery and development of bevacizumab, an anti-VEGF antibody for treating cancer. Nat. Rev. Drug Discov..

[111] Ferrara N., Hillan K.J., Novotny W. (2005). Bevacizumab (Avastin), a humanized anti-VEGF monoclonal antibody for cancer therapy. Biochem. Biophys. Res. Commun..

[112] Reshkin S.J., Bellizzi A., Caldeira S., Albarani V., Malanchi I., Poignee M., Alunni-Fabbroni M., Casavola V., Tommasino M. (2000). Na^+^/H^+^ exchanger-dependent intracellular alkalinization is an early event in malignant transformation and plays an essential role in the development of subsequent transformation-associated phenotypes. FASEB J..

[113] Boron W.F. (2004). Regulation of intracellular pH. Adv. Physiol. Educ..

[114] Reshkin S.J., Greco M.R., Cardone R.A. (2014). Role of pHi, and proton transporters in oncogene-driven neoplastic transformation. Philos. Trans. R. Soc. Lond. B. Biol. Sci..

[115] Counillon L., Bouret Y., Marchiq I., Pouysségur J. (2016). Na^+^/H^+^ antiporter (NHE1) and lactate/H^+^ symporters (MCTs) in pH homeostasis and cancer metabolism. Biochim. Biophys. Acta.

[116] Cardone R.A., Casavola V., Reshkin S.J. (2005). The role of disturbed pH dynamics and the Na^+^/H^+^ exchanger in metastasis. Nat. Rev. Cancer.

[117] Cong D., Zhu W., Shi Y., Pointer K.B., Clark P.A., Shen H., Kuo J.S., Hu S., Sun D. (2014). Upregulation of NHE1 protein expression enables glioblastoma cells to escape TMZ-mediated toxicity via increased H+ extrusion, cell migration and survival. Carcinogenesis.

[118] Zheng G., Peng C., Jia X., Gu Y., Zhang Z., Deng Y., Wang C., Li N., Yin J., Liu X. (2015). ZEB1 transcriptionally regulated carbonic anhydrase 9 mediates the chemoresistance of tongue cancer via maintaining intracellular pH. Mol. Cancer.

[119] Gallagher F.A., Sladen H., Kettunen M.I., Serrao E.M., Rodrigues T.B., Wright A., Gill A.B., McGuire S., Booth T.C., Boren J. (2015). Carbonic Anhydrase Activity Monitored In Vivo by Hyperpolarized 13C-Magnetic Resonance Spectroscopy Demonstrates Its Importance for pH Regulation in Tumors. Cancer Res..

[120] Parks S.K., Pouyssegur J. (2015). The Na^+^/HCO_3_^−^ Co-Transporter SLC4A4 Plays a Role in Growth and Migration of Colon and Breast Cancer Cells. J. Cell. Physiol..

[121] Lee S., Axelsen T.V., Andersen A.P., Vahl P., Pedersen S.F., Boedtkjer E. (2016). Disrupting Na^+^, HCO_3_^−^-cotransporter NBCn1 (Slc4a7) delays murine breast cancer development. Oncogene.

[122] Gorbatenko A., Olesen C.W., Boedtkjer E., Pedersen S.F. (2014). Regulation and roles of bicarbonate transporters in cancer. Front. Physiol..

[123] Cardone R., Alfarouk K., Elliott R., Alqahtani S., Ahmed S., Aljarbou A., Greco M., Cannone S., Reshkin S. (2019). The Role of Sodium Hydrogen Exchanger 1 in Dysregulation of Proton Dynamics and Reprogramming of Cancer Metabolism as a Sequela. Int. J. Mol. Sci..

[124] White K.A., Grillo-Hill B.K., Barber D.L. (2017). Cancer cell behaviors mediated by dysregulated pH dynamics at a glance. J. Cell Sci..

[125] Webb B.A., Chimenti M., Jacobson M.P., Barber D.L. (2011). Dysregulated pH: A perfect storm for cancer progression. Nat. Rev. Cancer.

[126] Harguindey S., Orozco J.P., Alfarouk K.O., Devesa J. (2019). Hydrogen ion dynamics of cancer and a new molecular, biochemical and metabolic approach to the etiopathogenesis and treatment of brain malignancies. Int. J. Mol. Sci..

[127] Harguindey S., Alfarouk K., Orozco J.P., Hardonniere K., Stanciu D., Fais S., Devesa J. (2020). A New and Integral Approach to the Etiopathogenesis and Treatment of Breast Cancer Based upon Its Hydrogen Ion Dynamics. Int. J. Mol. Sci..

[128] Harguindey S., Reshkin S.J. (2017). The new pH-centric anticancer paradigm in Oncology and Medicine; SCB, 2017. Semin. Cancer Biol..

[129] Asgharzadeh M.R., Barar J., Pourseif M.M., Eskandani M., Niya M.J., Mashayekhi M.R., Omidi Y. (2017). Molecular machineries of pH dysregulation in tumor microenvironment: Potential targets for cancer therapy. BioImpacts.

[130] Persi E., Duran-Frigola M., Damaghi M., Roush W.R., Aloy P., Cleveland J.L., Gillies R.J., Ruppin E. (2018). Systems analysis of intracellular pH vulnerabilities for cancer therapy. Nat. Commun..

[131] Roger S., Gillet L., Le Guennec J.-Y., Besson P. (2015). Voltage-gated sodium channels and cancer: Is excitability their primary role?. Front. Pharmacol..

[132] Fraser S.P., Ozerlat-Gunduz I., Brackenbury W.J., Fitzgerald E.M., Campbell T.M., Coombes R.C., Djamgoz M.B.A. (2014). Regulation of voltage-gated sodium channel expression in cancer: Hormones, growth factors and auto-regulation. Philos. Trans. R. Soc. Lond. B. Biol. Sci..

[133] Xia J., Huang N., Huang H., Sun L., Dong S., Su J., Zhang J., Wang L., Lin L., Shi M. (2016). Voltage-gated sodium channel Na_v_1.7 promotes gastric cancer progression through MACC1-mediated upregulation of NHE1. Int. J. Cancer.

[134] Patel F., Brackenbury W.J. (2015). Dual roles of voltage-gated sodium channels in development and cancer. Int. J. Dev. Biol..

[135] Brackenbury W.J., Djamgoz M.B.A., Isom L.L. (2008). An Emerging Role for Voltage-Gated Na+ Channels in Cellular Migration: Regulation of Central Nervous System Development and Potentiation of Invasive Cancers. Neurosci..

[136] Onkal R., Djamgoz M.B.A. (2009). Molecular pharmacology of voltage-gated sodium channel expression in metastatic disease: Clinical potential of neonatal Nav1.5 in breast cancer. Eur. J. Pharmacol..

[137] Koltai T. (2015). Voltage-gated sodium channel as a target for metastatic risk reduction with re-purposed drugs. F1000Research.

[138] Mohammed F.H., Khajah M.A., Yang M., Brackenbury W.J., Luqmani Y.A. (2016). Blockade of voltage-gated sodium channels inhibits invasion of endocrine-resistant breast cancer cells. Int. J. Oncol..

[139] Blankenship J.E. (1976). Tetrodotoxin: From poison to powerful tool. Perspect. Biol. Med..

[140] Hanifin C.T., Gilly W.F. (2015). Evolutionary history of a complex adaptation: Tetrodotoxin resistance in salamanders. Evolution (N. Y.).

[141] Narahashi T., Moore J.W., Scott W.R. (1964). Tetrodotoxin Blockage of Sodium Conductance Increase in Lobster Giant Axons. J. Gen. Physiol..

[142] Hagen N.A., du Souich P., Lapointe B., Ong-Lam M., Dubuc B., Walde D., Love R., Ngoc A.H., Canadian Tetrodotoxin Study Group (2008). Tetrodotoxin for Moderate to Severe Cancer Pain: A Randomized, Double Blind, Parallel Design Multicenter Study. J. Pain Symptom Manag..

[143] Hagen N.A., Lapointe B., Ong-Lam M., Dubuc B., Walde D., Gagnon B., Love R., Goel R., Hawley P., Ngoc A.H. (2011). A multicentre open-label safety and efficacy study of tetrodotoxin for cancer pain. Curr. Oncol..

[144] Narahashi T. (2008). Tetrodotoxin: A brief history. Proc. Jpn. Acad. Ser. B. Phys. Biol. Sci..

[145] Yang M., Kozminski D.J., Wold L.A., Modak R., Calhoun J.D., Isom L.L., Brackenbury W.J. (2012). Therapeutic potential for phenytoin: Targeting Na_v_1.5 sodium channels to reduce migration and invasion in metastatic breast cancer. Breast Cancer Res. Treat..

[146] Shao J., Feng G. (2013). Selective killing effect of oxytetracycline, propafenone and metamizole on A549 or Hela cells. Chin. J. Cancer Res..

[147] Márián T., Szabó-Péli J., Németh E., Trón L., Friedlander E., Szabó A., Balkay L., Veress G., Krasznai Z. (2007). Na^+^/Ca^2+^ exchanger inhibitors modify the accumulation of tumor-diagnostic PET tracers in cancer cells. Eur. J. Pharm. Sci..

[148] Iwamoto T., Watanabe Y., Kita S., Blaustein M. (2008). Na^+^/Ca^2+^ Exchange Inhibitors: A New Class of Calcium Regulators. Cardiovasc. Hematol. Disord. Targets.

[149] Rodrigues T., Estevez G.N.N., Tersariol I.L.d.S. (2019). Na^+^/Ca^2+^ exchangers: Unexploited opportunities for cancer therapy?. Biochem. Pharmacol..

[150] Liskova V., Hudecova S., Lencesova L., Iuliano F., Sirova M., Ondrias K., Pastorekova S., Krizanova O. (2019). Type 1 Sodium Calcium Exchanger Forms a Complex with Carbonic Anhydrase IX and Via Reverse Mode Activity Contributes to pH Control in Hypoxic Tumors. Cancers.

[151] Harguindey S., Arranz J.L., Polo Orozco J.D., Rauch C., Fais S., Cardone R.A., Reshkin S.J. (2013). Cariporide and other new and powerful NHE1 inhibitors as potentially selective anticancer drugs-an integral molecular/biochemical/metabolic/clinical approach after one hundred years of cancer research. J. Transl. Med..

[152] Harguindey S., Stanciu D., Devesa J., Alfarouk K., Cardone R.A.R.A., Polo Orozco J.D.J.D., Devesa P., Rauch C., Orive G., Anitua E. (2017). Cellular acidification as a new approach to cancer treatment and to the understanding and therapeutics of neurodegenerative diseases. Semin. Cancer Biol..

[153] Mijatovic T., Dufrasne F., Kiss R. (2012). Na^+^/K^+^-ATPase and cancer. Pharm. Pat. Anal..

[154] Alevizopoulos K. (2018). Na^+^/K^+^ ATPase Inhibitors in Cancer. Curr. Drug Targets.

[155] Khajah M.A., Mathew P.M., Luqmani Y.A. (2018). Na^+^/K^+^ ATPase activity promotes invasion of endocrine resistant breast cancer cells. PLoS ONE.

[156] Jansson B. (1996). Potassium, sodium, and cancer: A review. J. Environ. Pathol. Toxicol. Oncol..

[157] Eil R., Vodnala S.K., Clever D., Klebanoff C.A., Sukumar M., Pan J.H., Palmer D.C., Gros A., Yamamoto T.N., Patel S.J. (2016). Ionic immune suppression within the tumour microenvironment limits T cell effector function. Nature.

[158] Reshkin S.J., Cardone R.A., Harguindey S. (2013). Na^+^-H^+^ exchanger, pH regulation and cancer. Recent Pat. Anticancer. Drug Discov..

[159] Stühmer W., Alves F., Hartung F., Zientkowska M., Pardo L.A. (2006). Potassium channels as tumour markers. FEBS Lett..

[160] Kunzelmann K. (2005). Ion Channels and Cancer. J. Membr. Biol..

[161] Pardo L.A., Contreras-Jurado C., Zientkowska M., Alves F., Stühmer W. (2005). Role of Voltage-gated Potassium Channels in Cancer. J. Membr. Biol..

[162] Pardo L.A. (2004). Voltage-Gated Potassium Channels in Cell Proliferation. Physiology.

[163] Zhou Q., Kwan H.-Y., Chan H.-C., Jiang J.-L., Tam S.-C., Yao X. (2003). Blockage of voltage-gated K+ channels inhibits adhesion and proliferation of hepatocarcinoma cells. Int. J. Mol. Med..

[164] Choi S.Y., Kim H.-R., Ryu P.D., Lee S.Y. (2017). Regulation of voltage-gated potassium channels attenuates resistance of side-population cells to gefitinib in the human lung cancer cell line NCI-H460. BMC Pharmacol. Toxicol..

[165] García-Quiroz J., Camacho J. (2011). Astemizole: An old anti-histamine as a new promising anti-cancer drug. Anticancer. Agents Med. Chem..

[166] Huang X., Jan L.Y. (2014). Targeting potassium channels in cancer. J. Cell Biol..

[167] Ma J., Hou Y., Xia J., Zhu X., Wang Z.P. (2018). Tumor suppressive role of rottlerin in cancer therapy. Am. J. Transl. Res..

[168] Asensio-López M.C., Soler F., Pascual-Figal D., Fernández-Belda F., Lax A. (2017). Doxorubicin-induced oxidative stress: The protective effect of nicorandil on HL-1 cardiomyocytes. PLoS ONE.

[169] Chen C.C., Hong H.J., Hao W.R., Cheng T.H., Liu J.C., Sung L.C. (2019). Nicorandil prevents doxorubicin-induced human umbilical vein endothelial cell apoptosis. Eur. J. Pharmacol..

[170] Mun A.-R., Lee S.-J., Kim G.-B., Kang H.-S., Kim J.-S., Kim S.-J. (2013). Fluoxetine-induced apoptosis in hepatocellular carcinoma cells. Anticancer Res..

[171] Chen W.-T., Hsu F.-T., Liu Y.-C., Chen C.-H., Hsu L.-C., Lin S.-S. (2019). Fluoxetine Induces Apoptosis through Extrinsic/Intrinsic Pathways and Inhibits ERK/NF-κB-Modulated Anti-Apoptotic and Invasive Potential in Hepatocellular Carcinoma Cells In Vitro. Int. J. Mol. Sci..

[172] Liu Y., Li T., Xu M., Che X., Jiang X. (2017). Fluoxetine enhances cellular chemosensitivity to Cisplatin in cervical cancer. Int. J. Clin. Exp. Med..

[173] Cui C., Merritt R., Fu L., Pan Z. (2017). Targeting calcium signaling in cancer therapy. Acta Pharm. Sin. B.

[174] Nicotera P., Orrenius S. (1998). The role of calcium in apoptosis. Cell Calcium.

[175] Pinton P., Giorgi C., Siviero R., Zecchini E., Rizzuto R. (2008). Calcium and apoptosis: ER-mitochondria Ca^2+^ transfer in the control of apoptosis. Oncogene.

[176] Garrido C., Galluzzi L., Brunet M., Puig P.E., Didelot C., Kroemer G. (2006). Mechanisms of cytochrome c release from mitochondria. Cell Death Differ..

[177] Andreyev A., Tamrakar P., Rosenthal R.E., Fiskum G. (2018). Calcium uptake and cytochrome c release from normal and ischemic brain mitochondria. Neurochem. Int..

[178] Macmanus J.P., Whitfield J.F. (1971). Cyclic AMP-mediated stimulation by calcium of thymocyte proliferation. Exp. Cell Res..

[179] Pinto M.C.X., Kihara A.H., Goulart V.A.M., Tonelli F.M.P., Gomes K.N., Ulrich H., Resende R.R. (2015). Calcium signaling and cell proliferation. Cell. Signal..

[180] Berridge M.J. (1995). Calcium signalling and cell proliferation. BioEssays.

[181] Munaron L., Antoniotti S., Lovisolo D. (2004). Intracellular calcium signals and control of cell proliferation: How many mechanisms?. J. Cell. Mol. Med..

[182] Lusche D.F., Wessels D., Soll D.R. (2009). The effects of extracellular calcium on motility, pseudopod and uropod formation, chemotaxis, and the cortical localization of myosin II in Dictyostelium discoideum. Cell Motil. Cytoskeleton.

[183] Wei C., Wang X., Zheng M., Cheng H. (2012). Calcium gradients underlying cell migration. Curr. Opin. Cell Biol..

[184] Tsai F.C., Kuo G.H., Chang S.W., Tsai P.J. (2015). Ca^2+^ signaling in cytoskeletal reorganization, cell migration, and cancer metastasis. Biomed. Res. Int..

[185] Kale V.P., Amin S.G., Pandey M.K. (2015). Targeting ion channels for cancer therapy by repurposing the approved drugs. Biochim. Biophys. Acta Biomembr..

[186] Latour I., Louw D.F., Beedle A.M., Hamid J., Sutherland G.R., Zamponi G.W. (2004). Expression of T-type calcium channel splice variants in human glioma. Glia.

[187] Gackière F., Bidaux G., Delcourt P., Van Coppenolle F., Katsogiannou M., Dewailly E., Bavencoffe A., Van Chuoï-Mariot M.T., Mauroy B., Prevarskaya N. (2008). CaV3.2 T-type calcium channels are involved in calcium-dependent secretion of neuroendocrine prostate cancer cells. J. Biol. Chem..

[188] Ohkubo T., Yamazaki J. (2012). T-type voltage-activated calcium channel Ca v3.1, but not Ca v3.2, is involved in the inhibition of proliferation and apoptosis in MCF-7 human breast cancer cells. Int. J. Oncol..

[189] Dziegielewska B., Gray L.S., Dziegielewski J. (2014). T-type calcium channels blockers as new tools in cancer therapies. Pflugers Arch. Eur. J. Physiol..

[190] Saito K., Uzawa K., Endo Y., Kato Y., Nakashima D., Ogawara K., Shiba M., Bukawa H., Yokoe H., Tanzawa H. (2006). Plasma membrane Ca^2+^ ATPase isoform 1 down-regulated in human oral cancer. Oncol. Rep..

[191] Aung C.S., Kruger W.A., Poronnik P., Roberts-Thomson S.J., Monteith G.R. (2007). Plasma membrane Ca^2+^-ATPase expression during colon cancer cell line differentiation. Biochem. Biophys. Res. Commun..

[192] Dang D., Rao R. (2016). Calcium-ATPases: Gene disorders and dysregulation in cancer. Biochim. Biophys. Acta Mol. Cell Res..

[193] Sánchez M.G., Sánchez A.M., Collado B., Malagarie-Cazenave S., Olea N., Carmena M.J., Prieto J.C., Díaz-Laviada I. (2005). Expression of the transient receptor potential vanilloid 1 (TRPV1) in LNCaP and PC-3 prostate cancer cells and in human prostate tissue. Eur. J. Pharmacol..

[194] Kalogris C., Caprodossi S., Amantini C., Lambertucci F., Nabissi M., Morelli M.B., Farfariello V., Filosa A., Emiliozzi M.C., Mammana G. (2010). Expression of transient receptor potential vanilloid-1 (TRPV1) in urothelial cancers of human bladder: Relation to clinicopathological and molecular parameters. Histopathology.

[195] Dalton W.S., Crowley J.J., Salmon S.S., Grogan T.M., Laufman L.R., Weiss G.R., Bonnet J.D. (1995). A phase III randomized study of oral verapamil as a chemosensitizer to reverse drug resistance in patients with refractory myeloma. A southwest oncology group study. Cancer.

[196] Krouse A.J., Gray L., Macdonald T., Mccray J. (2015). Repurposing and rescuing of mibefradil, an antihypertensive, for cancer: A case study. Assay Drug Dev. Technol..

[197] Edwards J.C., Kahl C.R. (2010). Chloride channels of intracellular membranes. FEBS Lett..

[198] Liu B., Billington C.K., Henry A.P., Bhaker S.K., Kheirallah A.K., Swan C., Hall I.P. (2018). Chloride intracellular channel 1 (CLIC1) contributes to modulation of cyclic AMP-activated whole-cell chloride currents in human bronchial epithelial cells. Physiol. Rep..

[199] Warton K., Tonini R., Fairlie W.D., Matthews J.M., Valenzuela S.M., Qiu M.R., Wu W.M., Pankhurst S., Bauskin A.R., Harrop S.J. (2002). Recombinant CLIC1 (NCC27) Assembles in Lipid Bilayers via a pH-dependent Two-state Process to Form Chloride Ion Channels with Identical Characteristics to Those Observed in Chinese Hamster Ovary Cells Expressing CLIC1. J. Biol. Chem..

[200] Averaimo S., Milton R.H., Duchen M.R., Mazzanti M. (2010). Chloride intracellular channel 1 (CLIC1): Sensor and effector during oxidative stress. FEBS Lett..

[201] Valenzuela S.M., Martin D.K., Por S.B., Robbins J.M., Warton K., Bootcov M.R., Schofield P.R., Campbell T.J., Breit S.N. (1997). Molecular cloning and expression of a chloride ion channel of cell nuclei. J. Biol. Chem..

[202] Berryman M., Bretscher A. (2000). Identification of a novel member of the chloride intracellular channel gene family (CLIC5) that associates with the actin cytoskeleton of placental microvilli. Mol. Biol. Cell.

[203] Harrop S.J., DeMaere M.Z., Fairlie W.D., Reztsova T., Valenzuela S.M., Mazzanti M., Tonini R., Qiu M.R., Jankova L., Warton K. (2001). Crystal Structure of a Soluble Form of the Intracellular Chloride Ion Channel CLIC1 (NCC27) at 1.4-Å Resolution. J. Biol. Chem..

[204] Xu Y., Zhu J., Hu X., Wang C., Lu D., Gong C., Yang J., Zong L. (2016). CLIC1 Inhibition Attenuates Vascular Inflammation, Oxidative Stress, and Endothelial Injury. PLoS ONE.

[205] Setti M., Osti D., Richichi C., Ortensi B., Del Bene M., Fornasari L., Beznoussenko G., Mironov A., Rappa G., Cuomo A. (2015). Extracellular vesicle-mediated transfer of CLIC1 protein is a novel mechanism for the regulation of glioblastoma growth. Oncotarget.

[206] Lu J., Dong Q., Zhang B., Wang X., Ye B., Zhang F., Song X., Gao G., Mu J., Wang Z. (2015). Chloride intracellular channel 1 (CLIC1) is activated and functions as an oncogene in pancreatic cancer. Med. Oncol..

[207] Wei X., Li J., Xie H., Wang H., Wang J., Zhang X., Zhuang R., Lu D., Ling Q., Zhou L. (2015). Chloride intracellular channel 1 participates in migration and invasion of hepatocellular carcinoma by targeting maspin. J. Gastroenterol. Hepatol..

[208] Ye Y., Yin M., Huang B., Wang Y., Li X., Lou G. (2015). CLIC1 a novel biomarker of intraperitoneal metastasis in serous epithelial ovarian cancer. Tumor Biol..

[209] Wang P., Zhang C., Yu P., Tang B., Liu T., Cui H., Xu J. (2012). Regulation of colon cancer cell migration and invasion by CLIC1-mediated RVD. Mol. Cell. Biochem..

[210] Wang J.-W., Peng S.-Y., Li J.-T., Wang Y., Zhang Z.-P., Cheng Y., Cheng D.-Q., Weng W.-H., Wu X.-S., Fei X.-Z. (2009). Identification of metastasis-associated proteins involved in gallbladder carcinoma metastasis by proteomic analysis and functional exploration of chloride intracellular channel 1. Cancer Lett..

[211] Jia N., Dong S., Zhao G., Gao H., Li X., Zhang H. (2016). CLIC1 overexpression is associated with poor prognosis in pancreatic ductal adenocarcinomas. J. Cancer Res. Ther..

[212] Ding Q., Li M., Wu X., Zhang L., Wu W., Ding Q., Weng H., Wang X., Liu Y. (2015). CLIC1 overexpression is associated with poor prognosis in gallbladder cancer. Tumor Biol..

[213] Wang L., He S., TU Y., Ji P., Zong J., Zhang J., Feng F., Zhao J., Zhang Y., Gao G. (2012). Elevated expression of chloride intracellular channel 1 is correlated with poor prognosis in human gliomas. J. Exp. Clin. Cancer Res..

[214] Liu Y., Wang Z., Li M., Ye Y., Xu Y., Zhang Y., Yuan R., Jin Y., Hao Y., Jiang L. (2017). Chloride intracellular channel 1 regulates the antineoplastic effects of metformin in gallbladder cancer cells. Cancer Sci..

[215] Suh K.S., Mutoh M., Gerdes M., Crutchley J.M., Mutoh T., Edwards L.E., Dumont R.A., Sodha P., Cheng C., Glick A. (2005). Antisense suppression of the chloride intracellular channel family induces apoptosis, enhances tumor necrosis factor α-induced apoptosis, and inhibits tumor growth. Cancer Res..

[216] Ma P.-F., Chen J.-Q., Wang Z., Liu J.-L., Li B.-P. (2012). Function of chloride intracellular channel 1 in gastric cancer cells. World J. Gastroenterol..

[217] Edwards J.C. (2006). The CLIC1 chloride channel is regulated by the cystic fibrosis transmembrane conductance regulator when expressed in Xenopus oocytes. J. Membr. Biol..

[218] Gritti M., Würth R., Angelini M., Barbieri F., Peretti M., Pizzi E., Pattarozzi A., Carra E., Sirito R., Daga A. (2014). Metformin repositioning as antitumoral agent: Selective antiproliferative effects in human glioblastoma stem cells, via inhibition of CLIC1-mediated ion current. Oncotarget.

[219] Hong S., Bi M., Wang L., Kang Z., Ling L., Zhao C. (2015). CLC-3 channels in cancer (Review). Oncol. Rep..

[220] Liu C.L., Shi G.P. (2019). Calcium-activated chloride channel regulator 1 (CLCA1): More than a regulator of chloride transport and mucus production. World Allergy Organ. J..

